# Use sonoelastography to predict the reparability of large-to-massive rotator cuff tears

**DOI:** 10.1097/MD.0000000000021139

**Published:** 2020-07-02

**Authors:** Yu-Hsuan Tseng, Wen-Yi Chou, Kuan-Ting Wu, Ching-Di Chang, Yi-Cun Chen, Yu-Chi Huang, Wei-Che Lin, Po-Cheng Chen

**Affiliations:** a Department of Physical Medicine and Rehabilitation; b Department of Orthopedic Surgery, Kaohsiung Chang Gung Memorial Hospital, Chang Gung University College of Medicine; c Medical Mechatronic Engineering Program, Cheng Shiu University; d Department of Diagnostic Radiology, Kaohsiung Chang Gung Memorial Hospital, Chang Gung University College of Medicine, Kaohsiung; e Department of Public Health, College of Medicine, National Cheng Kung University, Tainan, Taiwan.

**Keywords:** rotator cuff tears, surgery, ultrasound elastography

## Abstract

**Introduction:**

Most symptomatic large-to-massive rotator cuff tears (RCTs) should be operated, but the surgical reparability depended on the degree of rotator cuff muscle atrophy or fatty infiltration. The orthopedic surgeons will decide whether the teared stump is reparable during the surgery, but preoperative evaluation can be done by some assessment tools. Magnetic resonance imaging (MRI) was used in recent studies to predict the reparability of large-to-massive RCTs, but the clinical availability was not as good as ultrasound. We hypothesize that the ultrasound elastography can predict the reparability of large-to-massive RCTs.

**Methods:**

This is a prospective observational study and participants with large-to-massive RCTs who are going to have surgeries will be included. Out investigators will evaluate the shoulder passive range of motion (ROM) and strength of all participants. Participants’ degree of shoulder pain and activities of daily living (ADLs) will be assessed by American Shoulder and Elbow Surgeons (ASES) score. The ultrasound elastography will be used to evaluate the tissue quality of supraspinatus muscle and infraspinatus muscle. To test the reliability of the ultrasound elastography, two physicians will perform the ultrasound elastography independently and twenty participants will be selected for the reliability test. Besides, MRI will be used to evaluate the size of tear, the degree of tendon retraction, fatty infiltration of rotator cuff muscles, and muscle atrophy. Finally, the orthopedic surgeons will perform surgeries and decide whether the teared stump can be completely repaired intraoperatively. The primary analysis is the predictive validity of ultrasound elastography for the reparability of large-to-massive RCTs. Before the predictive validity of ultrasound elastography is measured, our investigators will assess the reliability of ultrasound elastography when administered to cases with large-to-massive RCTs, and we will check the correlations between the findings of ultrasound elastography and MRI.

**Discussion:**

The outcome will provide the evidence of ultrasound elastography for preoperative evaluation of large-to-massive RCTs. The relationships between the findings of ultrasound elastography and MRI will also be examined for further analysis.

**Trial registration:**

Clinicaltrials.gov NCT03682679. Date of Registration: 25 September 2018, https://clinicaltrials.gov/ct2/show/NCT03682679?cond=rotator+cuff&cntry=TW&draw=2&rank=1.

## 1 Introduction

Rotator cuff tears (RCTs) are clinical problems resulting in shoulder pain and weakness, especially when performing shoulder forward flexion and abduction to horizontal position.^[1,2]^ According to recent epidemiological studies, the prevalence of RCTs is increasing with age.^[3,4]^ In addition to age, dominant arm, overweight, tobacco use, and hypertension are also risk factors of RCTs in one systematic review with meta-analysis.^[5]^ Large-to-massive RCTs accounted for about 40% of all RCTs in one observational study.^[6]^ Most symptomatic large-to-massive RCTs should be operated to mitigate shoulder pain and improve shoulder function, but the surgical reparability depended on the degree of rotator cuff muscle atrophy or fatty infiltration. Large-to-massive RCTs sometimes can be repaired completely, but they were irreparable under poor tissue quality or severe muscle atrophy. It is important to evaluate whether the teared stump can be completely repaired because the prognosis of partial repair was worse than that of complete repair.^[7]^ The orthopedic surgeons will decide whether the teared stump can be pulled back to the anatomic footprint, which is defined as complete repair, intraoperatively. However, the possibility of surgical reparability can also be predicted preoperatively with the help of some assessment tools.

There were growing studies focusing on predicting the reparability of large-to-massive RCTs using magnetic resonance imaging (MRI), in which the degree of tear size, tendon retraction, and fatty infiltration of muscle layer were evaluated.^[7–9]^ Sugihara et al^[9]^ started to evaluate the tear size and signal change of massive RCTs by MRI, and irreparable cases were associated with larger tear size, thickness of supraspinatus muscle, or high signal intensity of infraspinatus muscle. Another study found that the reparability was related to lesser diameter of tears measured at sagittal or coronal planes of MRI.^[10]^ One recently published study combined Goutallier classification,^[11]^ tangent sign,^[12]^ and Patte classification^[13]^ to predict the reparability of massive RCTs,^[14]^ and the discrimination of this model was excellent.

Recently, clinicians used the ultrasound to predict the reparability of RCTs, which was found to be related to the area of tears and age.^[15]^ Although this study included not only cases with large-to-massive RCTs, it was still the pioneer in this field of research. Rosskopf et al^[16]^ applied ultrasound shear wave elastography (SWE) to study the shear wave velocity (SWV) of supraspinatus muscles under different situations, including normal, tendinopathy, partial-thickness tear, and full-thickness tear. This research also studied the correlations between SWV of supraspinatus muscles and MRI findings, and SWV was correlated to the degree of tendon retraction and muscle atrophy of rotator cuff shown on the MRI. However, most of cases in this research was only less severe RCTs or even rotator cuff tendinopathy, and only small proportion of severely fatty infiltration was observed.

The primary objective of our study is to predict the reparability of large-to-massive RCTs using ultrasound elastography. Before the predictive validity of ultrasound elastography is measured, our investigators will assess the reliability of ultrasound elastography when administered to cases with large-to-massive RCTs, and we will check the correlations between the findings of ultrasound elastography and MRI.

## 2 Methods

### 2.1 Study design and setting

This is a prospective observational study. Patients suspected with RCTs will be recruited from orthopedic outpatient departments at Kaohsiung Chang Gung Memorial Hospital. The orthopedic surgeons will arrange MRI or ultrasound to confirm the diagnosis of large-to-massive RCTs. If the patients want to join this research project, the orthopedic surgeons will introduce the patients to the research assistant.

### 2.2 Ethical considerations

This study was approved by the Institutional Review Board of Chang Gung Memorial Hospital (IRB No. 201800883B0). Participants will be informed about this study by the research assistant through written informed consent. All the collected data or medical records should be used by the research team under the agreement of the participants. The entry to this study is determined by the patients, and the treatment will not be affected by the decision of participation. Participants can also withdraw consent to this study without influence on the clinical care or eligibility to participate in other research.

### 2.3 Participants

#### 2.3.1 Inclusion criteria

Participants with large-to-massive RCTs ready for arthroscopic surgeries are enrolled. The definition of large-to-massive RCTs is one of the following:

(1) The diameter of tear is greater than or equal to 3 cm.^[17]^
(2) Two or more rotator cuff full-thickness tears.^[18]^

#### 2.3.2 Exclusion criteria

Any of the following:

(1) Rotator cuff partial-thickness tears or small-to-medium full-thickness tears.
(2) Severe acromioclavicular arthritis requiring distal clavicle resection.
(3) Severe glenohumeral arthritis (Hamada classification grade 3 or above).^[19]^
(4) Pseudoparalysis.^[20]^
(5) History of shoulder fracture.
(6) Absolute contraindication for MRI, such as Claustrophobia, placement of cardiac pacemakers, neurostimulators, syringe injection pumps, cochlear implants, or any metal implant.

### 2.4 Data sources and measurement

Demographic characteristics, such as age, sex, body height and weight, side of lesion, duration of symptoms, job, exercise habit, and having inflammatory arthritis or not, will be recorded at the beginning of this study on a record book. These data will be regularly backed up in a hard drive protected by a password. In order to protect the privacy of the participants, the identity will be encrypted with a research code and English abbreviation. This encryption will not display the name of the participant, the number of the identity card, and the address. All research information will be treated as strictly confidential. If the participants can understand and sign the informed consent, they can be enrolled into the study.

### 2.5 Quantitative variables

All the outcome measurements will be evaluated at most 6 months before the surgery. The outcomes include:

#### 2.5.1 Physical exams

(1) Shoulder passive range of motion (ROM) at forward flexion, extension, abduction, internal rotation, and external rotation, by a goniometer.
(2) Shoulder strength of forward flexion, extension, abduction, internal rotation, and external rotation, by a manual dynamometer.

#### 2.5.2 Questionnaire

American Shoulder and Elbow Surgeons (ASES) score^[21]^ to evaluate the participants’ degree of shoulder pain and activities of daily living (ADLs).

##### 2.5.2.1 Image

**Figure 1:**
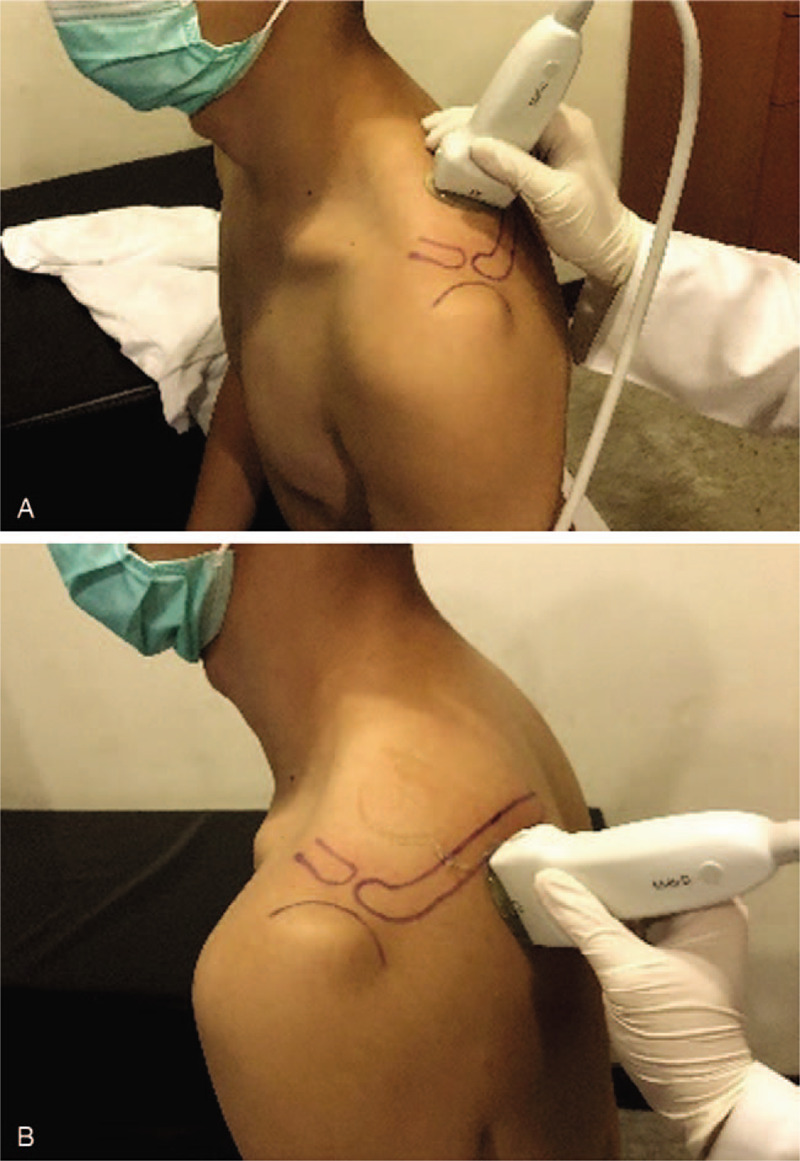
The scanning position of the participants using the ultrasound elastography. (A) The transducer position is perpendicular to the long axis of the supraspinatus muscle belly. (B) The transducer position is perpendicular to the long axis of the infraspinatus muscle belly.

(1) Ultrasound elastography: Physicians with at least 3-year experience of ultrasound will use Siemens Acuson S2000 ultrasound system (Siemens Healthcare, Erlangen, Germany) with a linear-array transducer with a bandwidth of 4 to 9 MHz to assess the middle third part of 2 rotator cuff muscles (supraspinatus muscle and infraspinatus muscle). The positioning of the ultrasound transducer is presented in Figure 1. The ultrasound transducer is perpendicular to the long axis of the muscle belly. The ultrasound elastography can evaluate the tissue quality by semiquantitative analysis and quantitative analysis.
  i. Semiquantitative analysis: Compressive ultrasound elastography will be used for the semiquantitative analysis. The pressure of the transducer on the skin should be controlled as the quality factor keeps at least 60 to achieve good color imaging.^[22]^ The region of interest (ROI) is located at the middle third part of each muscle. The software of ImageJ (Rasband, W.S., ImageJ, U. S. National Institutes of Health, Bethesda, MD, https://imagej.nih.gov/ij/, 1997–2018) is used to analyze the red pixels, green pixels, and blue pixels of the color histograms (each color pixels range: 0–255, with an accuracy of 0.1 pixel). The color pixels of each muscle will be the mean values of repeated measurements for 4 times.
  ii. Quantitative analysis: SWE will be used for quantitative analysis through the software of virtual touch tissue imaging quantification [VTIQ] (Siemens Healthcare, Erlangen, Germany). The ROI of each muscle will be separated into 4 quadrants. Our investigators will measure the SWV at the center of each quadrant for 4 times, and the SWV of each quadrant will be the mean value of these measurements. The unit of SWV is m/s with an accuracy of 0.01 m/s.
  iii. Test reliability: Twenty participants will be selected to test the reliability of color histograms of each color and SWV. Two physicians will perform the ultrasound elastography independently to test the inter-rater reliability. Besides, each physician will also repeat the exams at 2 separate time to test the intra-rater reliability.
(2) MRI: MRI will be performed on a 1.5T system (Signa Horizon LX, GE Healthcare) equipped with a standard shoulder surface coil. The shoulder MRI protocol is identical for all patients and consisted of axial proton-density-weighted fast spin-echo with fat suppression sequence, the sequences is performed above the level of the acromioclavicular joint down to below the axillary pouch with the following parameters: Coronal oblique proton-density-weighted with and without fat suppression, with axis parallel to the supraspinatus tendon, and sagittal oblique proton-density--weighted with and without fat suppression, with axis perpendicular to the coronal oblique axis, the fast spin-echo sequences are performed with the following parameters: TR ms/TE ms, 2700–4800 /25–40, echo-train length, 6; matrix, 256 × 256; field of view, 180 mm; section thickness, 2.5 mm with 2-mm gap. No IV or intraarticular gadolinium is administered. A musculoskeletal radiologist will read the images and evaluate the size of tear, the degree of tendon retraction (Patte classification^[13]^), fatty infiltration of rotator cuff muscles (Goutallier classification^[11]^), and muscle atrophy (tangent sign^[12]^).

### 2.6 Operative techniques

All patients are placed in the beach chair position. The surgeons will use a 30° angled arthroscope to establish the posterior entry point for intra-articular exploration at first, and the labrum, subscapularis tendon, and biceps brachii tendon are evaluated through the probe. If any tendon tear is observed, an anterior entry point will be established to repair the tendon. After the treatment of the intra-articular lesions, the arthroscope will be switched from the posterior entrance to the acromion, and the lateral entry point will be established, and the bursectomy will be performed from this entry point. If any subacromial spurs are found, acromioplasty will be performed to remove the spurs. Next, establish the posterolateral entry point and convert the arthroscope to this entry point to evaluate the rotator cuff lesions. We will use a ruler to measure the size of the rotator cuff tear (including the anterioposterior and mediolateral length of tear). The tendons will be released by electric burning to reduce the tension after suture. At the insertion of the rotator cuff tendons on the greater tuberosity, the surgeons will use a curette for debridement to increase the healing ability of the tendon. Finally, the shoulder is positioned at 60 degrees of abduction for rotator cuff tendon repair. After the discussion between two orthopedic surgeons, the surgery will be defined as “complete repair” or “partial repair” according to the intraoperative condition. If the ruptured tendons are reparable (or completely repaired), we will perform tendon repair using single-row or double-row sutures according to the size of tendon tear. If the ruptured tendon is irreparable (or partially repaired), we will perform partial repair to ensure that the infraspinatus tendon is reattached to the posterior side of the greater tuberosity and/or the subscapularis is attached to lesser tuberosity. If the ruptured tendon is flexible after releasing procedures, we will repair the tendon into the bone groove near the anatomical footprint and reinforce the structures with an acellular dermis. If the ruptured tendon is fully retracted without elasticity after releasing procedures, we will bridge the tendon to the anatomical footprint through an acellular dermis.

### 2.7 Statistical methods

We will use chi-square test or Fisher exact test to compare the difference of the categorical variables between the groups with complete repair and with partial repair. The continuous variables will be compared between the two groups by Mann-Whitney *U* test. In order to exam the reliability of the outcomes of ultrasound elastography (including color histograms of each color and SWV), intraclass correlation coefficients (ICC) will be used to evaluate the inter-rater reliability and intra-rater reliability. When it comes to the correlational analysis of the findings of ultrasound elastography and MRI, Spearman's rank correlation is used for 2 continuous variables, while Cramer's V correlation is used for one continuous variable versus one nominal variable. The predictive validity of ultrasound elastography for the reparability of large-to-massive RCTs will be analyzed by receiver operating characteristic (ROC) analysis to calculate the area under the curve (AUC). Finally, we will perform a logistic regression for the associations between the reparability and risk factors (such as age, sex, body mass index (BMI), inflammatory arthritis, the findings of ultrasound elastography, the findings of MRI). The statistical analysis will be performed using Stata 15 (StataCorp. 2017. Stata Statistical Software: Release 15. College Station, TX: StataCorp LLC.). A *P* value less than .05 will be defined as statistically significant.

### 2.8 Sample size estimation

The sample size estimation for a ROC analysis will based on a research by Hanley et al.^[23]^ According to Iagulli et al,^[24]^ about half of large-to-massive RCTs can be completely repaired, so the ratio of partial repair cases to complete repair cases is supposed to be 1. Under the assumption of (1) type I error = 0.05 (2) type II error = 0.2 (3) acceptable discrimination is 0.7, the sample size should be 62 participants. The estimated sample size will be corrected to 78 participants in consideration of 20% drop-out rate.

## 3 Discussion

In this study, we will inspect the reliability of ultrasound elastography when applied for preoperative evaluation of large-to-massive RCTs. The relationships between the findings of ultrasound elastography and MRI will also be examined. The degree of muscle atrophy and fatty infiltration of large-to-massive RCTs is more severe than other types of rotator cuff injury. Because the previous study by Rosskopf et al^[16]^ did not focus on the subgroups of large-to-massive RCTs, our study can confirm the application of ultrasound elastography in such cases. Besides, further ROC analysis and logistic regressions can help us to build a predictive model for reparability of large-to-massive RCTs preoperatively. Although similar study had been done by MRI,^[14]^ our study used ultrasound elastography to construct the predictive model for the sake of better availability than MRI. The outcomes of this study may help the clinicians for the preoperative prepare.

## Acknowledgments

We would also like to thank Ying-ping Wang, Hui-Hsin Tso, and Dun-jie Hu for their assistance for this research.

## Author contributions

All authors read and approved the final manuscript. PCC and KTW contributed to study design, development of recruitment pathways, ethical and governance approvals. YCH and WCL contributed to preparing the funding application and study design. WYC and CDC contributed to recruitment of participants and image interpretations. YHT and YCC contributed to manuscript preparation.
